# Mechanistic insights into HipA‐mediated bacterial persistence: Role of initial cellular states and toxin expression kinetics

**DOI:** 10.1002/mlf2.70095

**Published:** 2026-09-16

**Authors:** Jing Zhao, Yixiao Xiong, Hongxia Zhao, Jin Wang, Xiaona Fang

**Affiliations:** ^1^ School of Chemistry Northeast Normal University Changchun China; ^2^ State Key Laboratory of Electroanalytical Chemistry, Changchun Institute of Applied Chemistry Chinese Academy of Sciences Changchun China; ^3^ School of Applied Chemistry and Engineering University of Science and Technology of China Hefei China; ^4^ Department of Chemistry and Physics State University of New York at Stony Brook Stony Brook New York USA

**Keywords:** accumulation rate, growth phase, HipA, persistence, RelA

## Abstract

Persistent bacteria subpopulations showing multidrug tolerance pose significant challenges to antibiotic therapy. While the HipBA toxin–antitoxin (TA) system is known to play a role in persister formation, existing studies predominantly focus on its kinase activity and intracellular regulatory pathways, with limited attention to the environmental or conditional factors. In this study, we administered controlled induction of HipA across varied growth conditions in *Escherichia coli*, establishing that conditional constraints fundamentally modulate HipA's capacity to induce persistence. We demonstrated that cells in the lag phase, rather than in the exponential or stationary phase, were more likely to enter the persistent state upon HipA upregulation. Moreover, our results indicated that the accumulation rate of HipA, rather than the absolute HipA level, was a crucial determinant of persister frequency. Finally, we confirmed the essential role of RelA in HipA‐mediated formation of persistent bacteria. These findings highlight the importance of previously unexplored conditional factors and expression kinetics in HipA‐mediated persistence, providing mechanistic insights that link toxin kinetics, growth phase regulation, and stringent response signaling in bacterial antibiotic tolerance.

## Impact statement

Bacterial persisters survive lethal antibiotics and cause chronic infections. While the toxin HipA is a known driver of this survival, we discovered that its impact depends heavily on timing and speed. Surprisingly, bacteria are most prone to entering this protected state during their initial recovery phase (lag phase), rather than when fully dormant. Furthermore, the rate at which the toxin accumulates, rather than the total amount, determines survival. These findings reveal a “kinetic trap” that bacteria use to escape treatment, offering new targets for fighting recurrent infections.

## INTRODUCTION

As antibiotics are frequently used in therapeutic settings, bacterial resistance and the associated challenges have progressively increased^1^. Bacterial resistance primarily arises from genetic mutations, leading to a significant increase in the minimum inhibitory concentration (MIC)^2^. In addition to resistance, the presence of persistent bacteria further protects them from antibiotic therapy without acquiring genetic resistance. Persisters, first identified by Joseph Bigger, represent the survival of a small subgroup within bacterial populations exposed to lethal doses of antibiotics by entering a dormant state^3^, ^4^. Once antibiotics are removed, these bacteria can resume normal growth and reproduction, posing a significant challenge to effective antimicrobial therapy^5^, ^6^. Current research indicates that the presence of persistent bacteria may be a critical factor contributing to the difficulties associated with eradicating chronic diseases such as tuberculosis^7^, ^8^, cystic fibrosis^9^, and urinary tract infections^10^. Moreover, prolonged antibiotic treatment may accelerate the emergence of drug‐resistant bacteria due to the presence of persistent cells^11^. Therefore, comprehensive investigation into the mechanisms underlying the formation of persistent bacteria is essential for identifying potential therapeutic targets to control chronic and recurrent infections.

Currently, research into the mechanisms underlying persistent bacteria formation has identified numerous significant physiological processes and associated genes, including the toxin–antitoxin system^12^, the stringent response^13^, ^14^, the SOS response^15^, energy metabolism^16^, and quorum‐sensing systems^17^, ^18^. Among these mechanisms, toxin–antitoxin (TA) systems have garnered the most attention for their role in facilitating the formation of persisters. The gene *hipA* (high‐persistence gene A) in *Escherichia coli* was the first gene identified to be directly associated with the formation of persistent bacteria^19^, and its *hipA7* allele has been demonstrated to enhance the capacity for persistent bacteria formation by approximately 1000‐fold^20^, ^21^. As a component of the *hipBA* type II toxin–antitoxin system^22^, HipA promotes the development of persistent bacteria by phosphorylating Ser239, the active site of glutamyl tRNA synthetase^23^, ^24^. This phosphorylation results in the accumulation of uncharged glutamyl tRNA within the cell. The presence of this uncharged tRNA at the A site of the ribosome activates the (p)ppGpp signaling protein through RelA, which drives the cell into a dormant state^25^, ^26^. Furthermore, HipA overexpression inhibits protein synthesis, resulting in cell division arrest and increased resistance to β‐lactam and fluoroquinolone antibiotics^27^. The toxic effects of HipA can be neutralized by the antitoxin HipB^28^, thereby facilitating normal cellular activities. However, HipB is inherently less stable and can be degraded by Lon protease, which releases the active toxin HipA, leading to growth arrest and persistence. This process plays a significant role in the formation of bacterial persistence^29^, ^30^. Single‐cell observations have revealed a threshold in HipA expression that triggers bacterial persistence^2^, ^31^. It has been demonstrated that HipA overexpression increases persistence levels, with the toxin protein HipA playing a vital role in the formation of bacterial persisters through multiple pathways^32^, ^33^.

It is widely recognized that cells in the stationary phase are more likely to enter a persistent state^34^. A comparison of the persistence levels of HipA7 and HipA reveals that during the early stationary phase, the persistence level of HipA7 is 1000 times greater than that of the wild‐type strain. However, the frequency of persisters in the wild‐type strain increases dramatically with prolonged incubation time, ultimately reaching the level of the HipA7 mutant in the late stationary phase^35^. In other words, the frequency of persisters is influenced by the culture conditions of the inoculated bacteria, particularly the age of bacteria. However, there is currently no evidence to demonstrate whether the persistence of bacteria mediated by an increase in active HipA depends on cell growth status. Meanwhile, both the chromosomal allele mutation HipA7 and the overexpression of exogenous HipA can increase the persistence level of bacteria, raising another question: will the expression kinetics of HipA affect the formation of persistent bacteria?

To address these questions, we compared bacterial persistence rates under varying HipA expression patterns and conditions across different growth stages. Our findings indicated that the upregulation of HipA significantly increased the fraction of persister cells during the lag phase, while the impact on the persistence rate during the exponential phase was less pronounced. Furthermore, we discovered that the expression pattern of HipA is crucial for the formation of persistent bacteria, with the accumulation rate of HipA directly influencing the probability of persister cell formation within the same growth phase. Finally, we established the necessity of RelA in HipA‐mediated persister cell formation through control experiments utilizing *relA* knockout strains.

## RESULTS

### Construction and function of the toxin protein overexpression system

The hipBA toxin–antitoxin system is one of the most extensively studied Type II TA systems in bacteria, especially in *E. coli*. It consists of two genes: *hipB* and *hipA*, which encode the antitoxin protein HipB and the toxin protein HipA, respectively. Structural analyses have revealed that HipA forms a homodimer in conjunction with HipB, effectively repressing the transcription of the hipBA operon^36^. In a nonstressed growth environment, the antitoxin HipB neutralizes the toxic effects of HipA. Conversely, under stress conditions such as antibiotic exposure or nutrient limitation, the equilibrium between HipA and HipB can become disrupted, resulting in an increase in free HipA levels. This increase in intracellular free HipA slows down cell growth and has been shown to enhance the likelihood of persistent bacteria formation^37^, ^38^.

**Figure 1:**
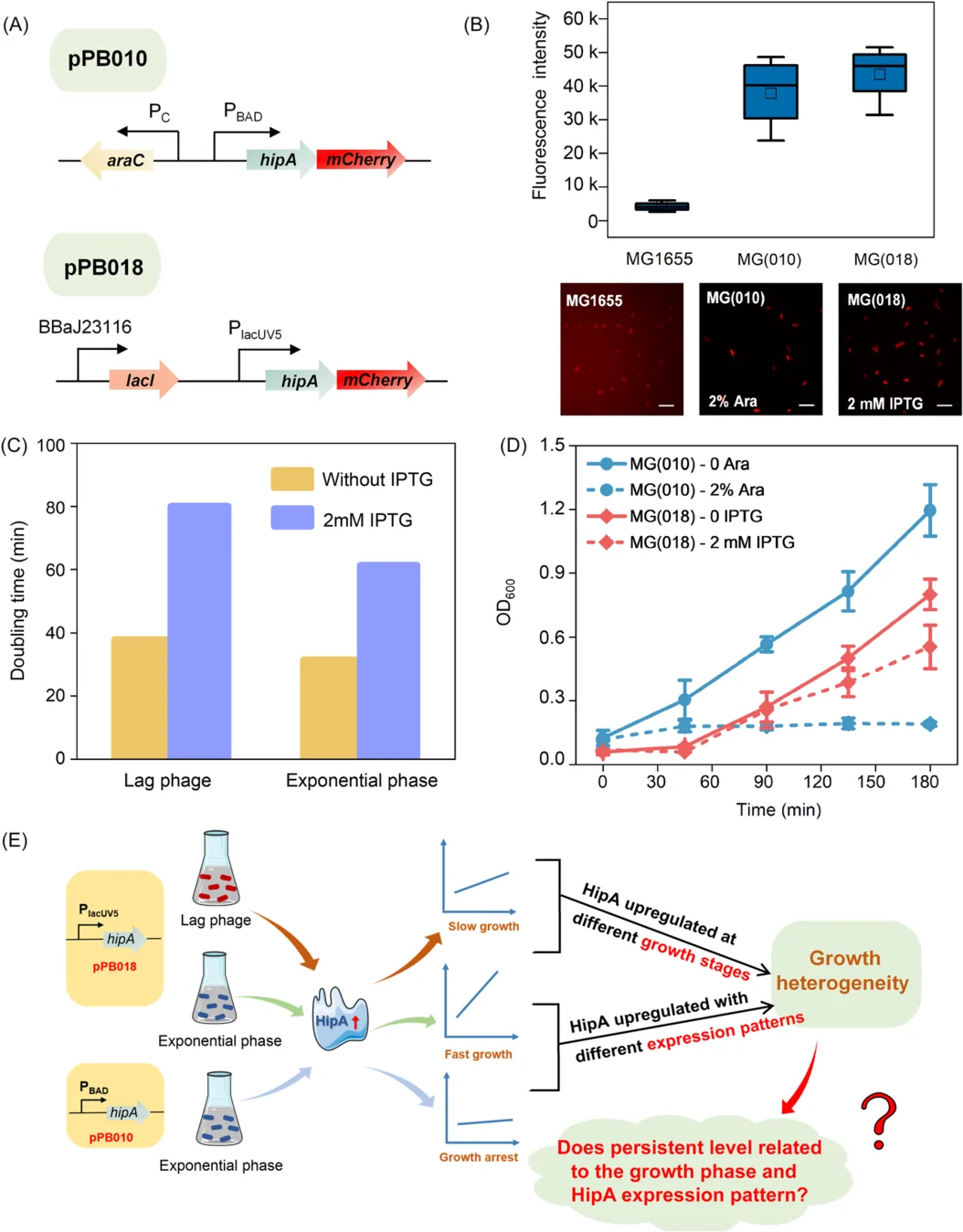
Characterization of HipA‐mCherry expression and its impact on bacterial growth under different regulatory systems. (A) Schematic representation of two different HipA expression systems: MG1655(pPB018) contains a constitutive BBaJ23116 promoter driving *lacI* expression and the P_lac_ _UV5_ system, inducible by IPTG, and MG1655(pPB010) contains the araC‐P_BAD_ system, inducible by arabinose (Ara). (B) Analysis of HipA‐mCherry expression. Upper panel: Quantitative analysis of fluorescence intensity in strains MG1655 (control), MG(010) (the MG1655 strain harboring plasmid pPB010) induced with 2% Ara for 3 h, and MG(018) (the MG1655 strain harboring plasmid harboring pPB018) induced with 2 mM IPTG for 3 h. Box plots show the distribution and average fluorescence intensity (AU). Lower panels: Representative fluorescence microscopy images confirming the expression of HipA‐mCherry in the corresponding strains. The control strain (MG1655) shows minimal background fluorescence, while induced MG(010) and MG(018) show distinct red fluorescence. Scale bar, 10 μm. (C) Impact of HipA expression on bacterial growth. Doubling time of the MG1655(pPB018) strain during the lag phase and the exponential phase as the initial state in the presence or absence of 2 mM IPTG induction is shown. The doubling time is calculated based on the overall OD_600_ measurement values of the growth curves. For each curve, the exponential growth phase was determined, and the doubling time was calculated using the relationship between OD_600_ and time. Data show that IPTG‐induced HipA expression significantly prolonged the doubling time. (D) Growth curves of MG1655(pPB010) induced by 2% Ara, MG1655(pPB018) induced by 2 mM IPTG, and the corresponding control strains without inducers. Data represent the mean of at least three independent experiments; error bars indicate the standard deviation (SD). (E) A conceptual model illustrating the experimental hypothesis. The model explores the relationship between persister formation and factors such as the growth phase (slow growth in the lag phase vs. rapid growth in the exponential phase) and HipA expression patterns. P_C_, constitutive promoter.

In this study, we aimed to investigate the persister fraction at varying levels of HipA expression by constructing plasmids that utilize the inducible promoters P_lacUV5_ and P_BAD_ for the regulation of HipA expression. To facilitate detection, *hipA* was fused with the gene encoding red fluorescent protein mCherry to generate the plasmids pPB018 and pPB010 (Tables S1 and S2). This fusion enabled us to monitor and quantify HipA expression through fluorescent detection (Figure 1A). The plasmids pPB010 and pPB018 were transformed into the wild‐type strain MG1655 to generate strains of MG1655(pPB010) and MG1655(pPB018). Consequently, we could regulate and quantify the expression level of intracellular HipA by adjusting the concentration of inducers arabinose (Ara) or isopropyl‐beta‐D‐thiogalactopyranoside (IPTG)^39^, ^40^.

To validate the functions of the two systems, we conducted induction experiments under varying conditions. Initially, we cultured the two bacterial strains to the exponential phase, induced for 3 h using either 2% Ara (pPB010) or 2 mM IPTG (pPB018). The induced cells were then examined microscopically, and their fluorescence intensity was quantified from the captured fluorescence images (Figure 1B). Meanwhile, flow cytometry analysis also corroborated the increase in fluorescence intensity upon induction (Figure S1), demonstrating that the expression level of the fusion protein HipA‐mCherry can be effectively modulated through induction. To directly confirm the expression and integrity of the HipA‐mCherry fusion protein under both induction systems, we performed Western blot analysis. As shown in Figure S2, a single, specific band of approximately 80 kDa was detected in the respective lanes using an anti‐mCherry antibody. The comparable intensity of the bands between the Ara‐ and IPTG‐ induced samples demonstrated similar expression levels of the fusion protein. We then investigated the impact of HipA overexpression on cell growth. The inducer IPTG was added to MG1655(pPB018) bacterial cells at different growth stages to monitor the impact of HipA on cellular growth. Figure 1C shows the doubling times of cell division when HipA was upregulated in cells initially at the lag phase and the exponential phase. The results indicated that upregulation of the toxin protein HipA can slow down cell growth, but its impact varied significantly between different growth phases, with cells in the lag phase being more substantially disrupted, suggesting that the sensitivity of cells to HipA toxicity is growth stage‐specific. To confirm that the overexpression of HipA, rather than mCherry, mediated growth inhibition, control plasmid pPB008 was constructed by removing the *hipA* gene from pPB010 (Figure S3A). The growth curves of MG1655(pPB008) demonstrated that Ara induction resulted in minimal changes in the cell growth rate in the absence of HipA (Figure S3B), thereby confirming that the overexpression of HipA impacts cell growth. Additionally, we compared the two strains of MG1655(pPB018) and MG1655(pPB010), which use distinct promoter systems to induce HipA expression. Figure 1D displays the growth curves of both strains when HipA expression was induced during the exponential phase. The results revealed that MG1655(pPB018) (with P_lacUV5_ promoter) showed only a slight growth defect by HipA upregulation (red dashed curve), whereas MG1655(pPB010) (with the P_BAD_ promoter) showed almost complete growth arrest (blue dashed curve) under 2% Ara induction, although the quantitative analysis in Figure 1B indicated comparable HipA protein levels between the two strains. Additionally, the observed differences in growth between uninduced MG1655(pPB010) and MG1655(pPB018) can be attributed to the basal expression of HipA due to promoter leakage from P_lacUV5_, which was demonstrated by the slightly higher basal expression level of MG1655(pPB018), as shown in Figure S1. This discrepancy indicates that expression kinetics determined by promoter characteristics, rather than the absolute toxin concentration, dominated the physiological effects. These findings reveal a dual regulatory mechanism in which the synergistic effect of cellular growth status and toxin expression pattern jointly determined the HipA‐mediated growth inhibition. This raises a crucial question: does this growth inhibition create a “kinetic trap” that facilitates the formation of persister cells? (Figure 1E).

### The initial state of the cell is crucial for the formation of toxin protein‐mediated persister

**Figure 2:**
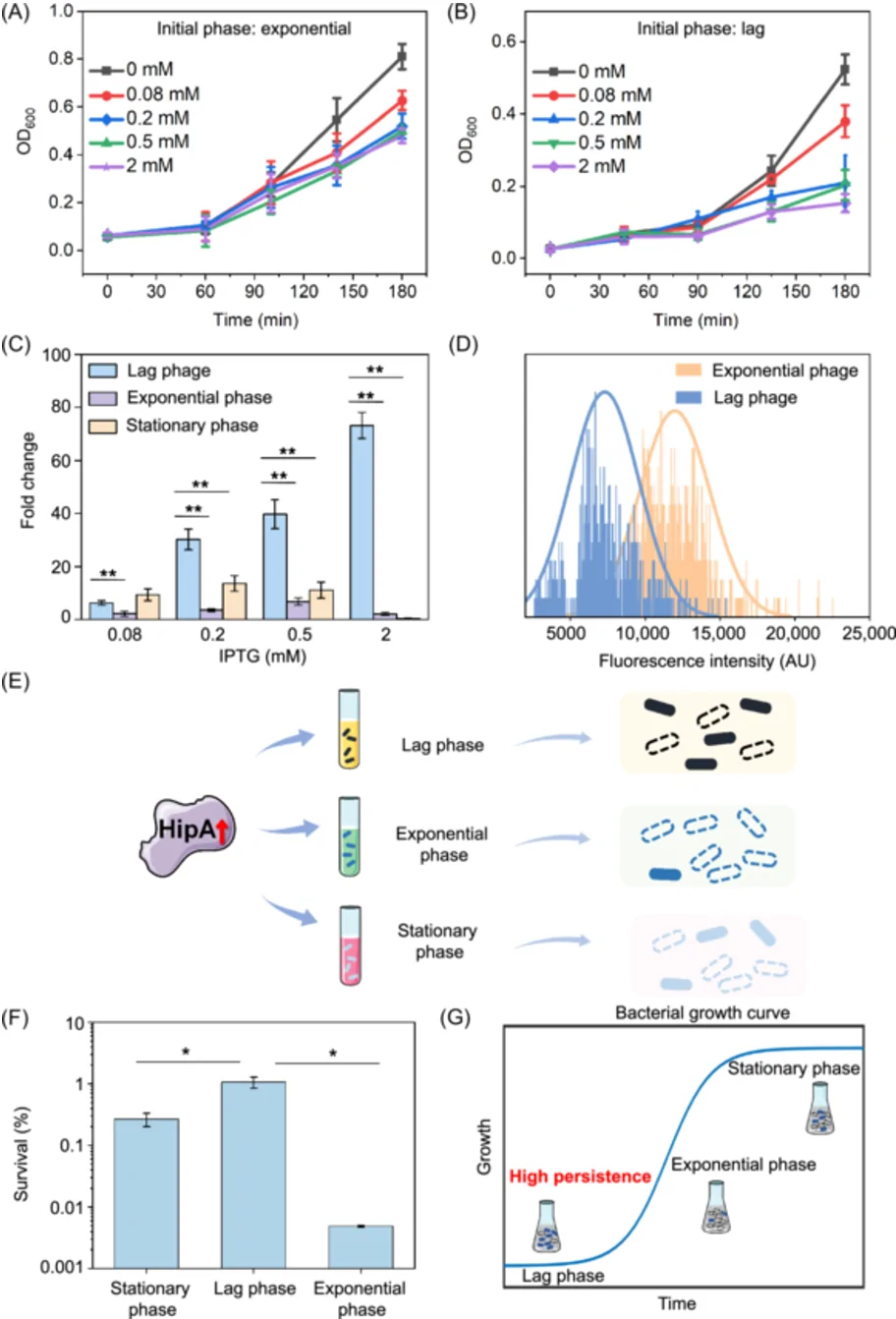
Induction results of MG1655(pPB018) in different initial states. (A, B) Growth curves (OD_600_) of the MG1655(pPB018) strain under different IPTG concentrations, with the exponential phase (A) and the lag phase (B) as the initial state. (C) Fold change in the survival rate (persister frequency) of the MG1655(pPB018) strain when IPTG treatment was administered during the lag, exponential, or stationary phase, compared to the control strain without IPTG. The survival rate was obtained after treatment with 500 μg/ml ampicillin for 12 h. Data are presented as mean ± SD (*n* = 3), **p < 0.01. (D) Fluorescence intensity distributions of cells induced by 0.08 mM IPTG, with the exponential phase and the lag phase as the initial state. (E) Schematic representation of HipA upregulation during various bacterial growth phases and its effects on bacterial antibiotic tolerance. Cells in the lag phase are more likely to enter the persistent state. (F) Survival of wild‐type MG1655 cells in the lag, stationary, and exponential phases, highlighting the highest survival in the lag phase. The survival rate was obtained after treatment with 500 μg/ml ampicillin for 12 h. Data are presented as mean ± SD (*n* = 3), *p < 0.05. (G) Schematic illustration of persistence levels across growth phases. Cells at various growth stages show differing tolerance to antibiotics; during the lag phase, they show increased tolerance, enabling them to enter the persistent state. Statistical significance was determined by the one‐way ANOVA (C, F). ANOVA, analysis of variance.

To investigate whether the persistent bacteria induced by HipA overexpression are influenced by growth status, we initially compared the growth rates of MG1655(pPB018) across varying levels of HipA expression. The overnight culture was diluted 1:1000 into fresh medium and incubated at 37°C for 3 h until reaching the exponential phase (OD_600_ ~ 0.1). Subsequently, various concentrations of IPTG were added for induction, and the OD values after induction were monitored to plot growth curves, as shown in Figure 2A. It can be observed that as the IPTG concentration increased, the elevated expression of HipA resulted in a decrease in the cell growth rate, although the extent of this reduction was not substantial. Following this, bacterial cells were treated with 500 μg/ml of ampicillin for 12 h, and their survival rates were determined by colony‐forming unit (CFU) count on Luria‐Bertani (LB) agar plates after antibiotic removal (Table S3). To analyze the impact of HipA upregulation on survival rates, we calculated the fold change in survival rates of cells induced with varying IPTG concentrations compared to those without induction (Figure 2C, purple bars). The results revealed that for cells in the exponential growth phase, despite a decrease in the growth rate due to the overexpression of HipA, the change in the survival rate was minimal, increasing by less than 10 times (with 2.13 ± 0.55 times for 2 mM IPTG induction and other conditions, see Table S4). This suggests that a certain degree of HipA expression upregulation does not substantially enhance the persister fraction. This phenomenon may be linked to the metabolic and vitality status of the cells at the time of HipA upregulation. Cells in the exponential phase show vigorous metabolism, and the growth slowdown caused by HipA overexpression did not result in a transition to a dormant state.

To further validate this hypothesis, we conducted a control experiment in which overnight cultured bacteria were diluted 1:100 into fresh LB medium, and various concentrations of IPTG were added simultaneously for induction. The OD_600_ values at different induction times were monitored, and growth curves were plotted (Figure 2B). Under these conditions, the initial state of the cells when HipA began to be induced by IPTG remained in the lag phase. Throughout the entire induction process, the growth rate of the cells varied significantly with IPTG‐induced HipA overexpression, showing a clear gradient change corresponding to the concentration of IPTG. We also calculated the survival rate and its fold change, which are presented in Tables S3 and S4 and Figure 2C (blue bars). The results indicated that HipA upregulation increased the persister fraction by 10~70 times (with 72.29 ± 6.55 times for 2 mM IPTG; see Table S4), significantly higher than that of cells induced from the exponential phase. Concurrently, stationary‐phase cells following overnight culture were also induced for HipA overexpression, and their survival rate was assessed after antibiotic treatment (Figure 2C, orange bars). Their persistence levels were also significantly higher than those of cells in the exponential phase.

To confirm that the difference in persister fraction under the varying conditions is indeed attributed to the cell states at the start of induction, rather than differences in HipA expression levels, we quantified the expression of HipA‐mCherry by measuring mCherry fluorescence on the microscope. Figure 2D illustrates the distributions of mCherry fluorescence induced by 0.08 mM IPTG for 3 h, with the lag phase and the exponential phase as the initial induction states. Notably, the fluorescence intensity was significantly higher in cells that began in the exponential phase compared to those in the lag phase. Similar results were obtained under 0.2 mM IPTG induction (Figure S4), indicating that cells in the exponential phase with higher HipA expression showed lower antibiotic tolerance than those in the lag phase with lower expression. This suggests that for cells in the lag phase, a slight upregulation of HipA can lead to a significant increase in the persister fraction. In contrast, for cells in the exponential phase, even a substantial increase in HipA levels results in only a minor improvement in persister fraction. This indicates that the formation of persistent bacteria mediated by HipA upregulation is not solely related to its inhibition on cell growth but is also closely linked to the cellular state at the time of HipA upregulation (Figure 2E).

Contrary to the prevailing notion that bacteria in the stationary phase show higher persistence, we surprisingly observed that *E. coli* cells were most susceptible to entering a persistent state during the lag phase, following a transition from nutrient‐depleted to nutrient‐rich conditions. This observation challenges previous reports suggesting a higher persistent fraction in stationary‐phase cells. To further validate our findings, we cultured the MG1655 cells (devoid of the HipA overexpression plasmid) to exponential, stationary, and lag phases, then treated them with ampicillin, and calculated their survival rates (Figure 2F). The results indicated that cells in the lag phase showed the highest survival rate (200 times that of exponential‐phase cells and 5 times that of stationary‐phase cells). These data further support our hypothesis that cells in the lag phase (or those undergoing abrupt nutrient shifts) are more prone to enter a persistent state (Figure 2G), possibly because cells in this state are more susceptible to changes under stress, leading to phenotypic heterogeneity. These findings propose a potentially novel mechanism for persistent bacteria formation: the initial state of the cells may serve as a crucial factor contributing to persistent bacteria formation, particularly those in the lag phase, which may be critical in promoting persistence.

### The accumulation rate of HipA protein influences the formation of persistent bacteria

**Figure 3:**
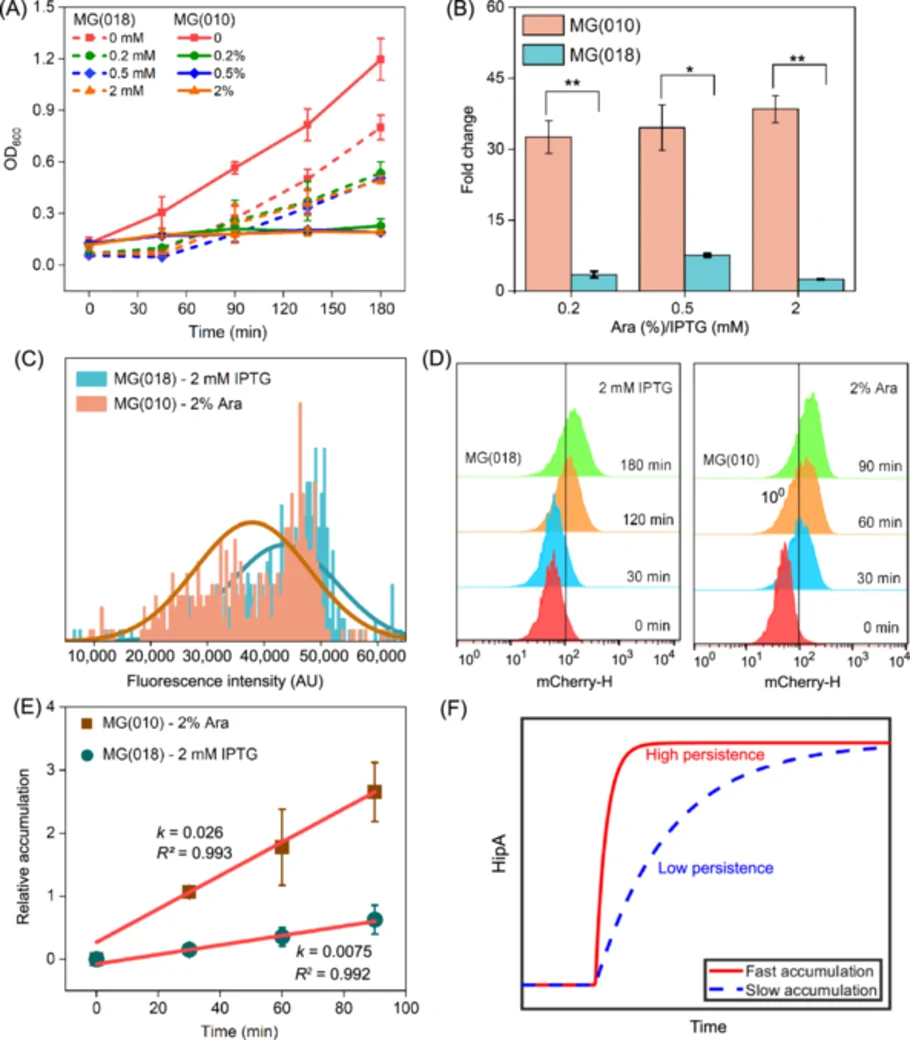
Induction results of MG1655(pPB010)/MG1655(pPB018) in the exponential phase as the initial state. (A) Growth curves of MG1655(pPB010) (induced by Ara, 0%–2%) and MG1655(pPB018) (induced by IPTG, 0–2 mM) with the exponential phase as the initial state. Data represent the mean of at least three independent experiments; error bars indicate SD. (B) Fold change in the survival rate of MG1655(pPB010) and MG1655(pPB018) strains after induction with different concentrations of inducers compared to the control strain without inducers. The survival rate was obtained after treatment with 500 μg/ml ampicillin for 12 h. Data are presented as mean ± SD (*n* = 3). Statistical significance was determined by the one‐way ANOVA test (0.2 mM, *p* = 0.004; 0.5 mM, *p* = 0.01; 2 mM, *p* = 0.002). (C) mCherry fluorescence distribution of MG1655(pPB010) induced with 2% Ara and MG1655(pPB018) induced with 2 mM IPTG for 3 h. (D) Dynamic changes in fluorescence intensity over induction time for MG1655(pPB010) and MG1655(pPB018) strains analyzed by flow cytometry. mCherry‑H refers to the pulse height value of the red fluorescence signal emitted by a single cell in the mCherry channel. (E) HipA accumulation in MG1655(pPB010) and MG1655(pPB018) strains over time measured by flow cytometry. The relative accumulation was calculated as (*I*–*I*_0_)/*I*_0_, where *I*_0_ represents the initial fluorescence intensity at *t* = 0. Linear regression analysis indicated the accumulation rates (*k*) and *R* ^2^ values for each strain, with a higher *k* for MG1655(pPB010), indicating faster HipA accumulation. Data represent the mean of at least three independent experiments; error bars indicate SD. (F) Schematic representation of the relationship between the HipA accumulation rate and persistence, with faster HipA accumulation leading to increased persistence. All measurements were performed in three replicates.

To further explore the formation mechanism of persistent bacteria, we examined whether HipA‐mediated persister formation is also influenced by factors other than the initial state. Based on the difference in the promoters P_lac‐UV5_ and P_BAD_ depicted in Figure 1A, ^39^, as well as the observed variations in their impact on the cell growth rate following induction, as shown in Figure 1D, we aim to further investigate whether this differential growth effect influences the formation of persistent bacteria. In our previous study, we demonstrated that cells in the lag phase were more prone to transition into the persistent stage. However, for the pPB010 system, we observed that when cells in the lag phase were induced with Ara, they completely failed to resume growth (Figure 3A), thereby hindering further investigation into bacterial persistence. Consequently, in this part, we opted to culture the cells to the exponential phase before inducing HipA overexpression, in order to examine the effect of HipA expression on the formation of persister bacteria. The growth curves presented in Figure 3A revealed significant differences between the two strains following induction with various inducers. Notably, the addition of inducer Ara to MG1655(pPB010) resulted in nearly stagnant growth, as determined by OD_600_‐based growth curves, with no observable variation in the growth rate across different inducer concentrations at 0.2%, 0.5%, and 2%. Conversely, MG1655(pPB018) showed consistent growth patterns as previously observed: the cells continued to grow after induction, albeit at a slightly reduced rate compared to the control strains without inducers. Subsequently, we compared survival rates in the two strains after antibiotic treatment in the presence of the inducer. Figure 3B illustrates the fold change in the survival rate between the two strains under HipA overexpression induction compared to the control group without inducers. The MG1655(pPB010) strain demonstrated a significantly enhanced capability to form persistent bacteria upon induction (30‐ to 40‐fold), whereas the MG1655(pPB018) strain showed minimal changes compared to the control (less than fivefold). Notably, while 0.2% Ara is commonly used for P_BAD_ promoter induction, we used 2% Ara based on initial optimization, showing higher expression levels from our construct at this concentration (Figure S3C). To address potential concerns about increased medium osmolarity affecting bacterial physiology in 2% Ara, we compared growth kinetics between 0.2% and 2% Ara induction for strains pPB010 and pPB008, observing no significant differences (Figures 3A and S3). Additionally, the fold change of survival rate under 0.2% Ara demonstrated consistent results with 2% induction (Figure 3B), confirming that the higher Ara concentration did not introduce physiological artifacts under our experimental conditions. Furthermore, upon examining fluorescence under the microscope and calculating the intensity, we observed that the distribution of HipA‐mCherry expression was similar in both strains post induction. However, the MG1655(pPB018) strain had a higher proportion of bacteria with intense fluorescence (Figure 3C). These results demonstrated that cells with identical initial growth states can show different persister fractions even under equivalent levels of toxin protein expression.

To clarify why similar levels of HipA resulted in significant differences in growth and survival rates under the control of different promoters, we compared the expression characteristics and efficiency of the two promoters. We measured the changes in fluorescence intensity over time during induction of the two strains via flow cytometry (Figure 3D). The results demonstrated that the P_BAD_ promoter (pPB010) showed rapid induction kinetics, accumulating to substantial levels within 60 min of 2% Ara induction. In contrast, the P_lac‐UV5_ promoter (pPB018) displayed significantly slower induction kinetics, requiring at least 180 min to reach full induction. Moreover, the induction of MG1655(pPB018) was strongly dependent on the inducer concentration. Cells induced by 0.2 mM IPTG for 180 min showed similar HipA‐mCherry expression to those induced by 2 mM IPTG for 120 min (Figure S5). Additionally, we found that the presence of the toxin protein HipA affected the expression levels of the two promoters. Using the flow cytometry data, we calculated the relative HipA accumulation rate over time (Figure 3E). The relative accumulation was calculated as (*I*–*I*_0_)/*I*_0_, where *I* represents the fluorescence intensity at each time point derived from the intensity data in Figure 3D and *I*_0_ represents the initial fluorescence intensity at *t* = 0. The HipA accumulation rate was determined from the slope of the linear regression curve during the initial stage of HipA increase, indicating a higher rate of *k* = 0.026 min^−1^ for MG1655(pPB010) and a much lower rate of *k* = 0.0075 min^−1^ for MG1655(pPB018).

These findings further elucidated another critical factor influencing HipA‐mediated persister formation: the rate of intracellular HipA accumulation. In other words, even under similar initial conditions where cells were induced at the same growth states and showed comparable levels of HipA expression, the process of upregulating HipA was also a crucial factor in the formation of persister cells. Cells were more likely to enter the persister states when HipA was rapidly upregulated and accumulated to an appropriate level in a short period. Conversely, when HipA was gradually and slowly upregulated to the same level over an extended period, the probability of persister cell formation decreased considerably (Figure 3F). This suggests that abrupt changes in pressure, such as toxin proteins, might be an indispensable factor in the formation of persistent bacteria.

### The nutrient environment affects the formation of persistent bacteria

**Figure 4:**
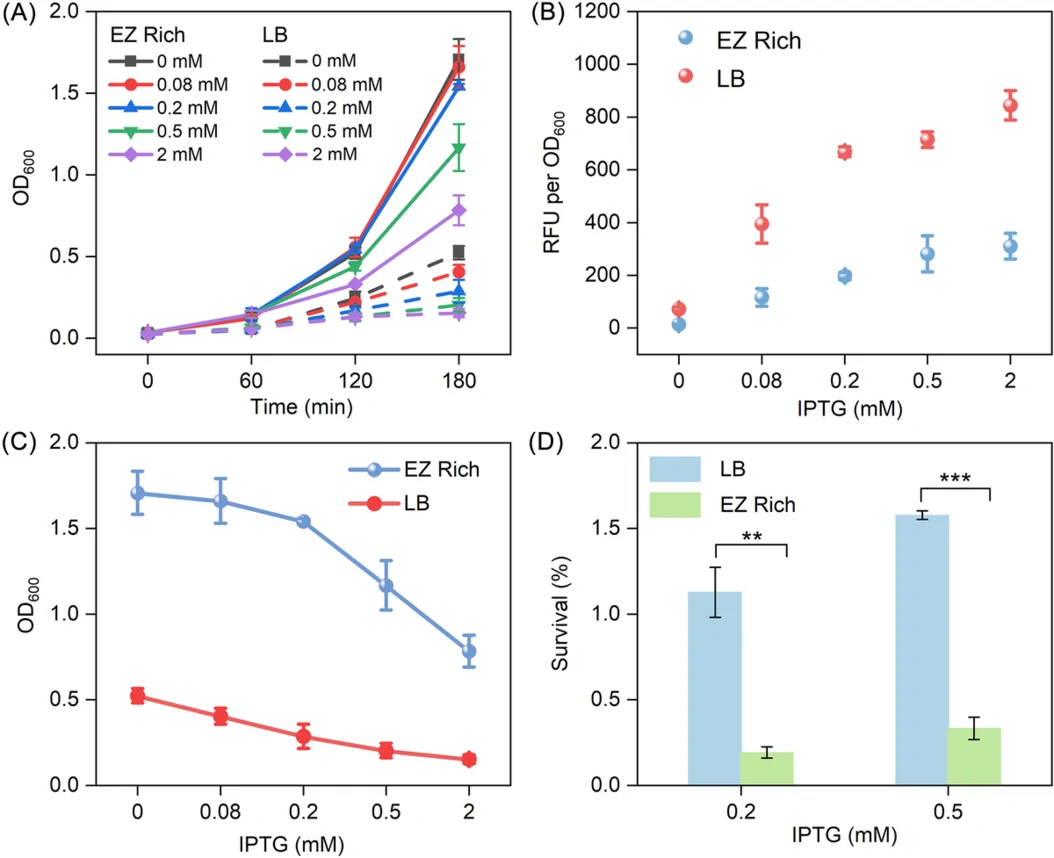
Induction results of MG1655(pPB018) in the culture medium of EZ‐Rich and LB media. (A) Growth curves of the MG1655(pPB018) strain in EZ‐Rich and LB media under different IPTG concentrations, with the lag phase as the initial state. (B) Mean fluorescence intensity of cells after 3 h of induction with varying IPTG concentrations. The relative fluorescence units (RFUs) were normalized by OD_600,_ with the lag phase as the initial state. (C) Final OD_600_ of the MG1655(pPB018) strain in EZ‐Rich and LB media after 3 h of induction with varying IPTG concentrations, with the lag phase as the initial state. (D) Survival of the MG1655(pPB018) strain in EZ‐Rich and LB media after 3 h of induction with 0.2 and 0.5 mM IPTG. The survival rate was obtained after treatment with 500 μg/ml ampicillin for 12 h. Data are presented as mean ± SD (*n* = 3). Statistical significance was determined by a one‐way ANOVA test (0.2 mM IPTG, *p* = 0.006; 0.5 mM IPTG, *p* = 0.0004). All measurements were performed in three replicates; error bars represent SD.

Next, we investigated the formation of persistent bacteria in different culture media with the lag phase as the initial state. LB and EZ‐Rich media were selected for experimentation, with EZ‐Rich being a nutrient‐rich medium. We examined the effect of HipA overexpression on the formation of persister in the MG1655(pPB018) strain under both culture media. Overnight cultured bacteria were diluted 1:100 into fresh IPTG supplied with LB or EZ‐Rich medium and cultured at 37°C. The growth curves in Figure 4A indicate that cells cultured in EZ‐Rich medium proliferated significantly faster than those in LB medium, regardless of whether HipA was overexpressed. Although the growth rate of induced cells was significantly reduced compared to non‐induced bacteria in EZ‐Rich medium, it still outperformed that observed in LB medium. To quantify the intracellular HipA‐mCherry levels under these two conditions, we measured the mean fluorescence intensity of the cells using a microplate reader and assessed single‐cell expression distribution via flow cytometry (Figures 4B and S6). It was evident that both single‐cell and overall fluorescence were stronger in the LB medium, indicating higher accumulation of toxin protein in the cells in LB medium. Moreover, as illustrated in the fluorescence microscopy images of cells induced with 0.08 mM IPTG in Figure S7, cells grown in LB medium appeared noticeably brighter than those in EZ‐Rich medium. As shown in Figure 4C, we plotted the curve of OD_600_ values after 3 h of induction with varying concentrations of IPTG. It can be observed that in EZ‐Rich medium, OD_600_ exceeded 1 (except for 2 mM IPTG induction), while they all remained below 0.5 in LB medium. This suggests that after induction, the cells in EZ‐Rich medium were closer to the stationary growth phase than those in LB. However, on examining the persistent fraction, we found that although a higher proportion of older cells was present in EZ‐Rich medium, their survival rate was much lower than that of cells in LB medium (Figure 4D). This is probably because the faster growth of cells in the nutrient‐rich EZ‐Rich medium leads to slower accumulation of HipA‐mCherry due to rapid cell division, which is the dilution effect. This observation further supports the conclusion drawn above: toxin protein‐induced persister formation is closely related to its accumulation rate.

### RelA is essential in HipA‐mediated persister formation

**Figure 5:**
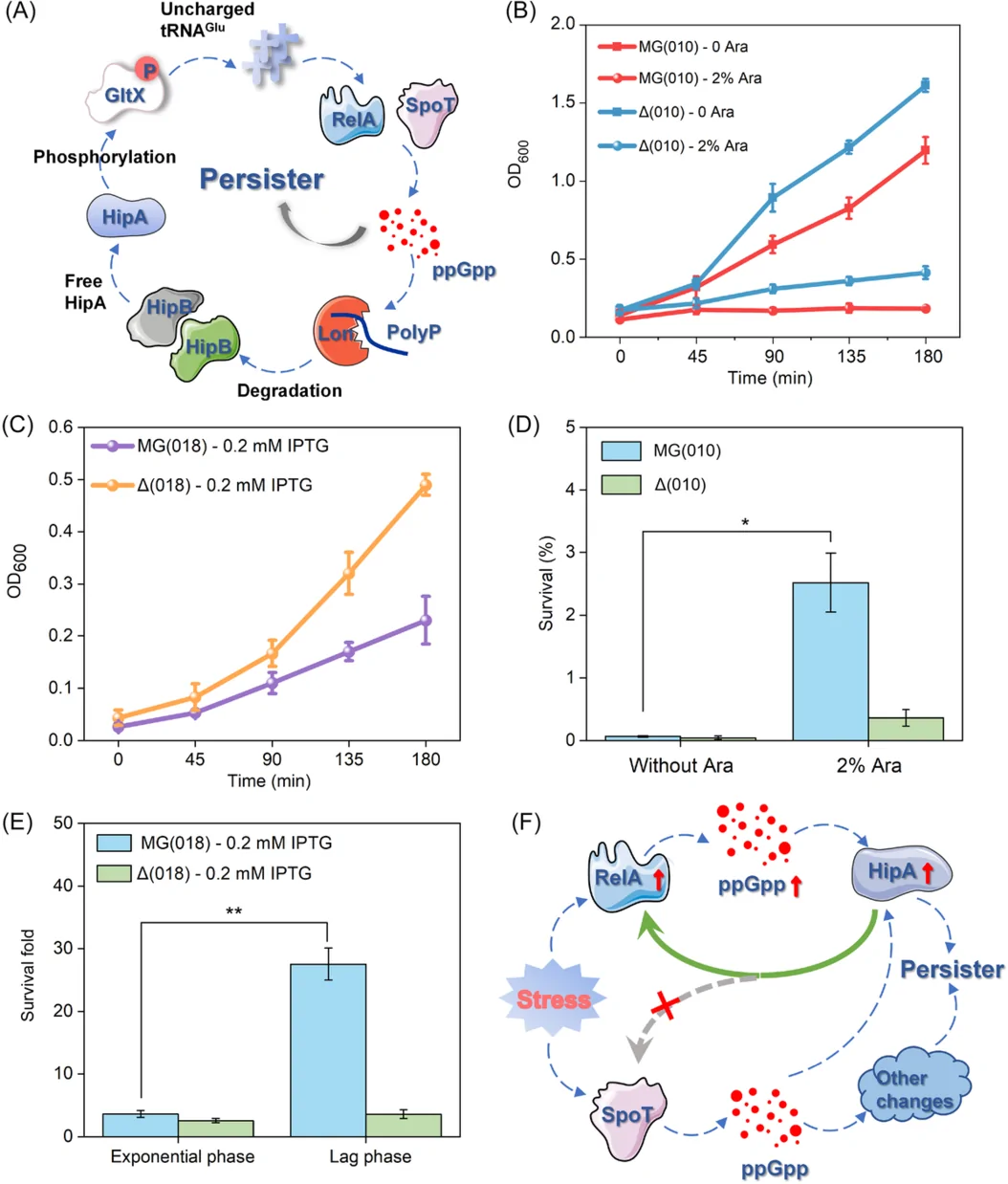
Role of RelA in HipA‐mediated persistence bacteria formation. (A) Schematic representation of the molecular mechanism involved in HipA‐mediated persister formation that has been reported^40^. (B) Growth curves of MG(010) and Δ(010) strains with or without 2% Ara induction. (C) Growth curves of MG(018) and Δ(018) strains under 0.2 mM IPTG induction. (D) Survival of MG(010) and Δ(010) strains with or without 2% Ara induction during the exponential phase as the initial state. The survival rate was obtained after treatment with 500 μg/ml ampicillin for 12 h. Data are presented as mean ± SD (*n* = 3). Statistical significance was determined by a one‐way ANOVA test (MG(010), *p* = 0.012; Δ(010), *p* = 0.05). (E) Survival fold of MG(018) and Δ(018) strains under 0.2 mM IPTG induction during the exponential and lag phases compared to the noninduced controls. The survival rate was obtained after treatment with 500 μg/ml ampicillin for 12 h. Data are presented as mean ± SD (*n* = 3). Statistical significance was determined by a one‐way ANOVA test (MG(018), *p* = 0.003; Δ(018), *p* = 0.082). (F) A molecular model explaining the role of RelA in the formation of HipA‐mediated persistent bacteria. In the absence of RelA, HipA cannot effectively activate SpoT‐mediated (p)ppGpp production. All measurements were performed in three replicates; error bars represented the standard deviation (SD).

Previous studies have suggested that HipA‐induced inactivation of glutamyl tRNA synthetase (GltX) leads to the accumulation of uncharged tRNA^Glu^ ^25^, which subsequently activates RelA and SpoT to produce the signaling molecule (p)ppGpp (guanosine tetra and penta‐phosphate). As a global regulator of bacterial stress responses, (p)ppGpp enhances the expression and activity of proteases like Lon, enabling it to degrade antitoxin HipB and release free HipA, thereby establishing a feedback loop that promotes bacterial persistence^40^ (Figure 5A). In our study, we investigated whether HipA‐mediated persistence is dependent on the *relA* gene in *E. coli*. We first knocked out the *relA* gene on the chromosome of MG1655, resulting in the MG1655::Δ*relA* strain. Then, the plasmids pPB018 and pPB010 were transferred into this strain, generating the strains MG1655::Δ*relA*(pPB018) and MG1655::Δ*relA*(pPB010), referred to as Δ(018) and Δ(010), respectively.

We first compared the growth rates of strains MG1655(pPB010) and Δ(010), as well as MG1655(pPB018) and Δ(018), when HipA expression was upregulated through induction. As shown in Figure 5B,C, the *relA* knockout strains grew faster than the wild‐type cells, indicating that the absence of RelA reduced the toxicity of HipA to the cells. Therefore, given the observed significant differences in growth rates, we speculate that RelA may be crucial in HipA‐mediated persister cell formation. To verify this conclusion, we compared the survival rates of strains MG1655(pPB010) and Δ(010). As shown in Figure 5D, the survival rates of MG1655(pPB010) and Δ(010) are quite similar under non‐inducing conditions. This aligns with previous reports, indicating that RelA‐mediated upregulation of ppGpp is not essential for the cells to enter a persistent state. Even in the absence of RelA, cells can still form persisters through alternative pathways, such as SpoT. However, we observed that when HipA was overexpressed under the strong promoter of P_BAD_ with induction, the survival rate of Δ(010) decreased by 85.6% compared to the wild‐type strain MG1655(pPB010). Combining this with the growth rate of HipA‐induced and non‐induced Δ(010) strains (Figure 5B), we found that although HipA overexpression in Δ(010) led to slower cell growth, the survival rate did not reach a level comparable to that of the MG1655(pPB010). This suggests that HipA overexpression in this strain might not activate the stringent response, and the slower growth might be simply due to the accumulation of uncharged tRNA^Glu^. We further compared the fold change of survival rates when HipA expression was upregulated during the exponential and lag phases for both the MG1655(pPB018) and Δ(018) strains. As illustrated in Figure 5E, the upregulation of HipA in exponentially growing MG1655(pPB018) cells led to a survival fold of 3.61 ± 0.55. In contrast, upregulation of HipA during the lag phase significantly increased survival to 28.34 ± 3.07, indicating an approximate 7.8‐fold enhancement relative to survival rates observed in exponential‐phase cells. In contrast, no such difference was observed in the *relA* knockout mutant Δ(018): upregulation of HipA in both exponential and lag phases did not lead to a significant increase in the survival rate. To genetically confirm the necessity of RelA in HipA‐mediated persistence, we conducted a genetic complementation experiment. A plasmid carrying the *relA* gene under its native chromosomal promoter was constructed and introduced into our Δ*relA* strains, generating the complementation strains Δ(018)+relA and Δ(010)+relA. Their survival rate was measured and compared with wild‐type and Δ*relA* strains. As the results show in Figure S8, the exogenous expression of *relA* from the plasmid fully restored persister levels to those comparable with the wild‐type strain. This result provides direct genetic evidence that the presence of RelA is specifically required for high HipA‐mediated persistence. This further demonstrates that the increase in the survival rate mediated by upregulated HipA expression is associated with the cell state, in which RelA plays a key role.

## DISCUSSION

In this study, we constructed mutant strains with exogenous overexpression of the toxin protein HipA and fused the red fluorescent protein mCherry with HipA to characterize the expression level of the toxin protein based on the fluorescence intensity of HipA‐mCherry. Meanwhile, we introduced inducible promoters upstream of HipA‐mCherry to regulate its expression in cells. By examining the growth rate of the cells, the expression level of HipA, and the persistence frequency, we initially revealed the relationship between the growth state of cells and the formation of persistent bacteria. Through comparisons of MG1655(pPB018) cells induced by IPTG at different growth stages, we found that the formation of HipA‐mediated persistent bacteria was closely related to the cellular state at the time of HipA expression upregulation. Cells in the exponential phase faced challenges in transitioning to the persistent state due to the overexpression of HipA. In contrast, cells in the lag phase demonstrated varying proportions of persistence depending on HipA levels. Notably, a higher fraction of persisters was observed in the lag phase cells of the wild‐type strain MG1655, indicating that these cells are more inclined to enter a persistent state. This finding is surprising and contradicts our previous understanding that cells in the stationary phase are more likely to transition into a persister state^34^, ^41^, suggesting that lag phase cells may show greater phenotypic heterogeneity. To further investigate factors affecting HipA‐mediated persister formation, we initiated HipA upregulation at the same growth phase within each comparison, using (1) the P_BAD_ and P_lac_ systems during the exponential phase and (2) the P_lac_ system under different culture conditions during the lag phase. The results demonstrate that the formation of HipA‐mediated persistent bacteria is influenced by the accumulation rate of free HipA protein. We speculate that there are two potential molecular mechanisms for this expression rate‐sensing phenomenon: First, HipA likely undergoes continuous degradation by cellular proteases like Lon and ClpXP. During slow accumulation, these proteases may efficiently clear HipA, preventing it from reaching effective concentrations. In contrast, rapid production could temporarily saturate this degradation system, allowing HipA to reach functional thresholds and trigger downstream effects. Second, the HipA‐mediated persister formation pathway involves a multi‐step cascade with potential threshold effects at each step. Rapid HipA accumulation may synchronize signals across these nodes, efficiently driving cells into persistence. These findings highlight the importance of both the growth phase and HipA accumulation dynamics in bacterial persistence, providing new insights into the complex regulatory mechanisms involved and potential avenues for developing novel anti‐persister strategies. Our results indicate that the homogeneous induction of HipA‐mCherry is sufficient to trigger the persister phenotype. However, the potential role of HipA expression heterogeneity in persister formation requires further exploration. In future work, single‐cell tracking and flow cytometry sorting techniques can be used to sort cell subpopulations based on variations in HipA‐mCherry expression levels, and to evaluate their survival rates under antibiotic stress. This approach is essential for validating the role of HipA heterogeneity in the formation of persistent bacteria. Finally, we also confirmed the role of RelA in the TA system. HipA toxicity may arise from two aspects: First, as a kinase, HipA phosphorylates Gltx, inhibiting the aminoacylation reaction and leading to the accumulation of uncharged tRNAGlu, which interferes with the synthesis of various proteins and slows down cell growth^26^. On the other hand, HipA triggers a stringent response and induces the upregulation of RelA expression, resulting in an increase in both RelA and ppGpp, causing cells to enter a dormant state. Previous studies have reported that in the absence of *relA*, bacteria can still enter a persister state through compensatory genes or pathways (like *spoT*)^42^, ^43^, ^44^. However, we demonstrated that HipA‐induced survival was markedly reduced in Δ*relA* strains and restored by *relA* complementation, suggesting that HipA‐mediated persister formation largely depends on RelA, whose loss was not fully compensated by endogenous alternative pathways, including those involving SpoT, under the conditions tested. In other words, the feedback loop formed by HipA and the ppGpp production from the stringent response is not entirely equivalent and closed; that is, ppGpp mediated by other pathways can lead to upregulation of HipA expression in the absence of RelA. However, HipA‐mediated ppGpp increase cannot occur without RelA. This may be because of the fact that HipA can significantly enhance the expression of RelA, but not SpoT. Therefore, RelA is essential for the intracellular ppGpp upregulation mediated by HipA. Therefore, we have revised our model of this pathway (Figure 5F). This finding contrasts with previous reports suggesting that *spoT* can substitute for *relA* in regulating ppGpp^45^. Consequently, we have included a discussion of the potential feedback loop that may modulate the persistence phenotype via *relA* and ppGpp.

Although we have drawn several critical conclusions regarding the involvement of toxin–antitoxin systems in the formation of persistent bacteria, the mechanisms underlying this process are extremely complex, involving numerous important physiological processes and related genes. To date, our investigations have focused solely on one aspect, namely, the toxin–antitoxin system, without considering other mechanisms that contribute to bacterial persistence, such as quorum sensing, which features significantly different cell‐to‐cell communication signals between the stationary and lag phases, and represents another key factor. Therefore, comprehensive research from diverse perspectives is essential to fully elucidate the mechanisms that govern the formation of persistent bacteria.

## MATERIALS AND METHODS

### Strains, plasmids, and medium

The bacterial strains and plasmids used in the experiments are listed in Table S1, and the primers used are listed in Table S2. The procedures for plasmid construction and gene knockout are described in the Supporting Information. *E. coli* strains were cultured in LB medium or EZ Rich Defined Medium at 37°C with shaking at 250 rpm. To maintain plasmid selection, chloramphenicol was added to the growth medium at a final concentration of 10 μg/ml as required.

### Toxicity detection

Strains containing the plasmid encoding the toxin protein HipA were pre‐cultured overnight and subsequently diluted 1000‐fold the following day. Different concentrations of l‐arabinose or IPTG were then added to induce the expression of HipA. After induction at 37°C for 12 h, the optical density at 600 nm (OD_600_) was measured to assess the effect of toxin protein HipA expression on cell growth.

### Fluorescence microscopy

Microscopy was performed using a Nikon Eclipse Ti inverted microscope system with a Plan Apo TIRF 100× Oil DIC H N2 objective and an Andor EMCCD camera. After culturing the cells to the appropriate density, inducers were added to induce the expression of toxin protein HipA for 3 h. mCherry fluorescence was measured under the excitation of a 561 nm laser. Exposure time and signal Electron Multiplier (EM) gain were adjusted based on the actual expression intensity and signal strength of the samples. Images were processed using NIS Element Image Analysis software (Nikon) and ImageJ (National Institutes of Health).

### Induction of toxin protein overexpression

For induction of toxin protein overexpression during the exponential phase, overnight pre‐cultured bacteria were diluted 1:1000 and transferred to LB or EZ‐Rich medium, and then cultured at 37°C for 3 h until the OD_600_ reached approximately 0.1. Different concentrations of inducers were added to induce the overexpression of the toxin protein HipA. Ara was added to strains harboring the pPB010 plasmid to induce HipA expression, while IPTG was added to strains containing the pPB018 plasmid. The cultures were then incubated at 37°C for an additional 3 h. For lag‐phase induction, the overnight pre‐cultured bacterial solution was diluted 1:100 and transferred to LB or EZ‐Rich medium. At the same time, the corresponding inducers were added to induce the overexpression of toxin protein HipA, and the cultures were incubated at 37°C for 3 h. For stationary‐phase induction, the inducers were directly added to the overnight precultured bacteria (16–18 h) to induce the overexpression of the toxin protein HipA for 3 h.

### Measurement of persistence

Persistence was assessed by determining the number of CFU per milliliter following exposure to ampicillin. Overnight cultures were diluted 100‐ or 1000‐fold in fresh medium containing chloramphenicol (10 μg/ml) to maintain plasmids (pPB010 or pPB018), and HipA expression was induced according to the experimental requirements. After induction, the inducers were washed away, and the bacteria were resuspended in fresh medium before being treated with 500 μg/ml ampicillin at 37°C for 12 h. For the determination of CFU, 1 ml aliquots were collected, and the cells were washed, serially diluted, and plated on LB agar plates. The persistence was calculated by dividing the number of CFU/ml in the culture after 12 h of incubation with the antibiotic by the number of CFU/ml in the culture before the addition of the antibiotic.

### Construction of mutant strains and complementation strains

To construct the *relA* deletion mutant, the entire coding region of *relA* was deleted from the wild‐type strain MG1655 via λ Red‐mediated gene replacement^46^ using primers P27 and P28 (see Table S2). This process generated the mutant strain MG1655Δ*relA*. For genetic complementation, the full‐length *relA* gene was PCR‐amplified from the MG1655 chromosome and subsequently ligated into the SpeI and BamHI sites of the plasmid pMO719 (laboratory stock), yielding the recombinant plasmid pPB020. This plasmid was then transformed into MG1655Δ*relA* to produce the complementation strain for functional assays.

## AUTHOR CONTRIBUTIONS

**Jing Zhao**: Data curation; formal analysis; investigation; validation; writing—original draft. **Yixiao Xiong**: Formal analysis; validation. **Hongxia Zhao**: Data curation; validation. **Jin Wang**: Conceptualization; methodology; writing—review and editing. **Xiaona Fang**: Conceptualization; funding acquisition; investigation; methodology; project administration; resources; supervision; visualization; writing—review and editing.

## ETHICS STATEMENT

This study did not involve human participants, animal experiments, or human tissues. All experiments involving bacterial cultivation and genetic modification were conducted in strict accordance with the biosafety regulations and guidelines of our institution. There are no foreseeable ethical issues in this study.

## CONFLICT OF INTERESTS

The authors declare no conflict of interests.

## Supporting information

mLife‐2025‐0133R‐Supplemental_figures_and_tables.

## Data Availability

The data supporting the findings of this study are available within the article and its supplementary materials.
