# Supplementary material for: Mechanistic insights into HipA‐mediated bacterial persistence: Role of initial cellular states and toxin expression kinetics
Source: mLife. 2026 Sep 16;5(5):651–64. doi: 10.1002/mlf2.70095 (PMC13579642; doi:10.1002/mlf2.70095)
Supplement: Supplementary file 1 [file MLF2-5-651-s001.docx]

**Supplementary Materials for**

**Mechanistic Insights into HipA-Mediated Bacterial Persistence: Role of Initial Cellular States and Toxin Expression Kinetics**

**Jing Zhao, Yixiao Xiong, Hongxia Zhao, Jin Wang, Xiaona Fang^*^**

**Supplemental Experimental Procedures**

**Strains and plasmids construction**

Plasmid pPB003 was constructed by PCR amplification of hipA from the chromosome of MG1655 using P1 and P2 primers and ligation into the NheI and KasI sites of the pMC002 (laboratory storage) plasmid.

pPB004 was constructed by PCR amplification of pLacO-hipA from pPB003 using P3 and P4 primers, then ligation into the pPB002 vector which was amplified from pPB002 (laboratory storage) using primers P5 and P6.

pPB005 was constructed by PCR amplification of araC-pBAD from pTKRED using P7 and P8 primers and ligation into the SacII and NheI sites of the pPB004 plasmid.

pPB006 was constructed by PCR amplification of hipAmCherry fusion protein from pPB003 using P9 and P10 primers and ligation into the HindIII and XbaI sites of the pPB005 plasmid.

pPB009 and pPB010 are based on the pPB006, where plasmid pPB009 is a splitting of the hipAmCherry fusion protein and plasmid pPB010 is a replacement of the linker fragment of the hipAmCherry fusion protein. pPB009 was constructed by PCR amplification of RBS-mCherry protein from pPB006 using P11 and P12 primers, and the vector with hipA-tga (termination codon) from pPB006 using the primer P13 and P14. The new linker of HipAmCherry fusion protein in pPB010 is GCCGGAACCTGATCC (Rotem et al., 2010) and comes from pBAD33hipA-mCherry the vector with hipA-linker was amplified by PCR on pPB006 plasmid with primers P15 and P16, the insert with mCherry (without start codon) was amplified by PCR on pPB006 with primers P17 and P18.

Plasmid pPB014 was obtained by replacing the hipA promoter of plasmid pPB010 from the pBAD system with the Plac-lacUV5 system, where the Plac-lacUV5 fragment was amplified from pPB013 (laboratory storage) by primers P19 and P20, and then ligation into the SacII and SphI sites of the pPB010 plasmid. The pPB014m plasmid is a point mutation plasmid for pPB014, changing the start codon of lacI from gtg to Atg. PCR amplification was performed with pPB014 template and primers P21 and P22.

pPB018 were base-expression optimized plasmid of pPB014, which further replaced the promoter of lacI based on pPB014m, using the BBaJ23 series of constitutive promoters. The promoters BBaJ23116 were obtained from pMT001 by PCR amplification using the primer P23-24; and the vectors were obtained from pPB014m by PCR amplification using the primers P25 and P26.

**Supplemental Figures**

**Figure S1.** The fluorescence intensity expression of the MG(010) and MG(018) strains after 3 hours of induction with 2% arabinose or 2mM IPTG was measured, with the inducer being added at OD_600_ ~ 0.1.

**Figure S2.** The Western Blot result diagram for expressing the HipA-mCherry fusion protein using 2% Ara to induce MG(010) or 2mM IPTG to induce MG(018). Left panel: Detection using anti-mCherry antibody shows the expression of the full-length HipA-mCherry fusion protein (expected size ~80 kDa). Lane 1&2: MG(010); Lane 3&4: MG(018). Internal control detection using anti-GAPDH antibody to verify equal protein loading. Right panel: Quantitative analysis of the mCherry signal was performed, and the results were normalized by GAPDH as a bar graph.

**Figure S3.** **(A)** Schematic diagram of plasmid pPB008, the control strain of plasmid pPB010 regulated by the arabinose promoter. **(B)** Growth curves of plasmid pPB008 induced by different concentrations of arabinose. After overnight pre-culture, the cells were diluted 1000 times and cultured until the OD_600_ reached 0.1. Then, different concentrations of arabinose were added for induction. Samples were taken at specific time points and the OD values were measured. The bars represent the mean of at least three independent experiments; the error bars indicate standard deviation(SD). **(C)** The fluorescence intensity of the MG(010) strains after 3 hours of induction with 0.2% and 2% arabinose.

**Figure S4.** Fluorescence intensity distributions of MG(018) induced by 0.2 mM IPTG, with the exponential phase and lag phase as the initial state.

**Figure S5.** **(A)** Change in fluorescence intensity of MG(018) over induction time at inducer concentrations of 0.2 mM IPTG with the exponential phase as the initial state, and the different colors represent different induction time. **(B)** Fluorescence intensity of MG(018) at different induction times and different induction concentrations. It can be observed that the fluorescence intensity at 120 minutes induced by 2 mM IPTG and at 180 minutes induced by 0.2 mM IPTG are basically the same.

**Figure S6.** Fluorescence intensity of MG(018) induced by 0.5 mM IPTG in different media with lag phase as the initial state.

**Figure S7.** Fluorescence expression of MG(018) in different media is illustrated, showing the fluorescence signal of mCherry under 0.08 mM IPTG induced HipA overexpression for 3 hours, captured by a fluorescence microscope under identical conditions. LB medium is displayed on the left side, while EZ-Rich medium is on the right side (Scale bar, 10 μm).

**Figure S8. (A**) The survival rate of MG(018), Δ(018) and Δ(018)+relA strains with 0.2mM IPTG induction during the lag phase as the initial state. (B) The survival rate of MG(010), Δ(010) and Δ(010)+relA strains with 2% Ara induction during the exponential phase as the initial state. The survival rate was obtained after treatment with 500 μg/ml ampicillin for 12 hours.

**Supplemental Tables**

**Table S1. Bacterial strains and plasmids.**

| **Strains and plasmids** | **Relevant characteristics** | **Source** |
| --- | --- | --- |
| MG1655 | WT E. coli K-12 | ATCC |
| MG1655Δ  pPB003  pPB004  pPB005  pPB006  pPB008  pPB009  pPB010  pPB014  pPB014m  pPB018  pPB020 | MG1655 ΔrelA::KanR  LacI-PLacO-hipAmCherry; GFP; KanR  PLacO-hipA; CmR  araC-P_BAD_-hipA; CmR  pPB005-hipAmCherry; CmR  pPB006-araC-P_BAD_-mCherry; CmR  pPB006-hipA-RBS-mCherry; CmR  pPB006-hipAmCherry new linker; CmR  pPB010-LacI-Plac lacUV5; CmR  pPB014-gtg-atg; CmR  pPB014m-BBaJ23116-LacI; CmR  relA; SmR | This work  This work  This work  This work  This work  This work  This work  This work  This work  This work  This work  This work |

**Table S2. Primers used in this study.**

| **Name** | **DNA sequence (5’-3’)** |
| --- | --- |
| P1 | tacg-**GCTAGC**-atgcctaaacttgtcacttgg |
| P2 | tata-**GGCGCC**-atacttccgctaccactgcc-cttactaccgtattctcggctt |
| P3 | attcc-**CCGCGG**-catggacctgtaggatcgt |
| P4 | gtcg-**TCTAGA**-gcatcacttactaccgtattctcggctt |
| P5 | gtcg-**TCTAGA**-gagtcagtgagcgaggaag |
| P6 | attcc-**CCGCGG**-gctcactcaaaggcggtaat |
| P7 | att-**CCGCGG**-ttatgacaacttgacggctac |
| P8 | gta-**GCTAGC**-tttttataacctccttagagctcg |
| P9 | gc-**TCTAGA**-cttacttatacagctcgtccat |
| P10 | ccc-**AAGCTT**-actgaagccagacttg |
| P11 | tc-**GGCCC**-ctaaggaggttataaaaagctagcatggtgagcaagggcgag |
| P12 | taat-**GTGCAC**-gaaccccccgttcag |
| P13 | gttc-**GTGCAC**-acagcccagctt |
| P14 | taat-**GGGCCC**-tcacttactaccgtattctcggctt |
| P15 | taat-**GGGCCC**-gagtcagtgagcgaggaagc |
| P16 | ctat-**TCTAGA**-*GCCGGAACCTGATCC*-cttactaccgtattctcggctt |
| P17 | ctat-**TCTAGA**-gtgagcaagggcgaggaggataaca |
| P18 | taat-**GGGCCC**-ttacttatacagctcgtccatgcc |
| P19 | tga-**CCGCGG**-ggttcttatggctcttgtatc |
| P20 | attc-**GCATGC**-tttttataagcgtcgactgtttcc |
| P21 | gagagtcaattcagggtggtgaatAtgaaaccagtaacgttatacgatgt |
| P22 | acatcgtataacgttactggtttcaTattcaccaccctgaattgactctc |
| P23 | tat-**CCGCGG**-tccatttgtcacgtcctgcac |
| P24 | gct-**AGATCT**-tctcctcctgctagcatagt |
| P25 | ggc-**AGATCT**-atgaaaccagtaacgttatacgatgtcg |
| P26  P27    P28 | tgt-**CCGCGG**-tacggttatccacagaatcag  TAGTTGCGATTTGCCGATTTCGGCAGGTCTGGTCCCTAAA  GGAGAGGACG-AAGGTCCAAACGGTGAAGGT  GTAGATACAGTATATATCAATCTACATTGTAGATACGAGCA  AATTTCGGC-CCTGTTATCCCTATTGGCTTC |

The bolded portion is the enzyme cleavage site, and the italicized portion in primer P16 is the ligated fragment of the HipAmCherry fusion protein.

**Table S3. The survival rate of MG(018) induced by IPTG at different phases and concentrations**

| **IPTG** | **Stationary phase induce** | **Lag phase induce** | **Exponential phase induce** |
| --- | --- | --- | --- |
| **0 mM** | 0.17%±0.014% | 0.043%±0.0057% | 0.043%±0.021% |
| **0.08 mM** | 1.45%±0.33% | 0.27%±0.086% | 0.085%±0.035% |
| **0.2 mM** | 2.26%±0.51% | 1.21%±0.028% | 0.15%±0.076% |
| **0.5 mM** | 1.59%±0.035% | 1.59%±0.014% | 0.28%±0.11% |
| **2 mM** | 0.05%±0.042% | 3.09%±0.13% | 0.088%±0.037% |

**Table S4. Survival fold of MG(018) induced by IPTG at different phases and concentrations**

| **IPTG** | **Stationary phase induce** | **Lag phase induce** | **Exponential phase induce** |
| --- | --- | --- | --- |
| **0.08 mM** | 8.67±2.67 | 6.18±1.19 | 2.19±0.93 |
| **0.2 mM** | 13.49±4.15 | 28.34±3.07 | 3.61±0.55 |
| **0.5 mM** | 9.42±0.99 | 37.27±4.58 | 6.79±1.35 |
| **2 mM** | 0.28±0.22 | 72.29±6.55 | 2.13±0.55 |

**Supplemental References**

Rotem, E., Loinger, A., Ronin, I., Levin-Reisman, I., Gabay, C., Shoresh, N., Biham, O., and Balaban, N.Q. (2010). Regulation of phenotypic variability by a threshold-based mechanism underlies bacterial persistence. Proceedings of the National Academy of Sciences of the United States of America *107*, 12541-12546.
